# Assessment of gallstone predictor: comparative analysis of ultrasonographic and biochemical parameters

**DOI:** 10.1186/1755-7682-6-17

**Published:** 2013-04-24

**Authors:** Hafiz Muhammad Aslam, Shafaq Saleem, Muhammad Muzzammil Edhi, Hiba Arshad Shaikh, Jehanzeb Daniel khan, Mehak Hafiz, Maria Saleem

**Affiliations:** 1 Dow Medical College, Dow University of Health sciences, Flat #14, 3rd floor, Rafiq Mansion, Cambell road, Off Arambagh, Karachi, Pakistan; 2 Liaquat National Medical College, Karachi University, Karachi, Pakistan; 3 Karachi Medical and Dental College, Karachi, Pakistan

**Keywords:** Gallstone, Alkaline phosphatase, Fatty liver

## Abstract

**Background:**

Gallstones represent a significant burden for health care systems worldwide and are one of the most common disorders presenting to emergency room. Ultrasonography, complete blood picture test and liver function tests are procedures of choice in suspected gallstones or biliary diseases. They are the most sensitive, specific, non-invasive and inexpensive tests for the detection of gallstones. Our main objective was to evaluate the relationship of ultrasonographic findings, hemolytic indices and liver function tests with gallstones.

**Methodology:**

It was a prospective study carried out in Civil Hospital Karachi (DUHS) and Liaquat National Hospital, two largest tertiary care hospitals of Karachi, Pakistan. Duration of the study was from July 2011 to October 2012. The study was carried out on diagnosed, pre-operative and symptomatic patients of cholelithiases. Exclusion criteria were patients of gallbladder and pancreatic carcinoma, emergency operations, patients having age <12 years and non-cooperative patients, who refused to give written consent for participation in the study. Total two tests were performed on each patient after diagnosis by ultrasonography. These were complete blood count and liver function tests. All the demographic data, laboratory findings and ultrasonographic features were noted in a pre-structured Performa. Sample size was calculated by using open-epidemiological sample size calculator prevalence (p) = 35%, d = 5%, and confidence interval (CI) 95% = 350. All the data was entered and analyzed through SPSS 19.

**Result:**

There were 454 diagnosed and pre-operative cases of gallstones present in the study. There were 120(26.4%) males and 334(73.6%) females, with a mean age of 42.80 ± 12.26 years. Most of the suspects had multiple stones 384 (84.5%) while few had single stones 70(15.4%). Fatty liver was found to be present in 144(31.7%) patients and 92(20.2%) had hepatomegaly. Splenomegaly was present in 16(3.5%) patients. Alkaline phosphatase was elevated in 186(41.0%) patients while SGPT was found to be raised in 160(35.2%). Blood urea nitrogen was found to be elevated in 186(41%) patients and serum creatinine was elevated in 46(10.1%) patients.

**Conclusion:**

In the light of findings it is recommend that all patients should go through the process of ultrasonography and all the biochemical parameters should be analyzed before surgery.

## Introduction

Gallstones represent a significant burden for health care systems worldwide and are one of the most common disorders presenting to emergency room [1]. It was once considered a disease of western world but due to changes in food pattern, now it is becoming an increasingly common cause of morbidity, leading to hospital admission in the developing world [2]. It is one of the most common disorders of gastrointestinal tract, affecting 10% people in western society [3]. Its occurrence in Asian population ranges from approximately 3-15% and in Pakistan incidence is about 4% and 14.2% in males and females respectively [4,5].

Gallstones can occur anywhere within the biliary tree [6]. They can occur due to the super saturation of bile, cholesterol precipitation, crystal formation, impaired gallbladder function and impaired of entero-hepatic circulation of bile acids [4]. There are various types of stones; mixed stones are cholesterol predominant, while black pigment stones consist of 7-10% calcium bilirubinate and brown pigment stones are formed as a result of infections which convert soluble bilirubin into insoluble state leading to formation of soft brown stones [7].

Ultrasonography is the procedure of choice in suspected gallstones or biliary diseases. It is the most sensitive, specific, non-invasive and inexpensive test for the detection of gallstones. Sensitivity is variable and dependent upon proficiency but in general it is highly specific and sensitive (>95% for stones <2 mm) [8,9]. A characteristic finding evaluated in ultrasound of gallbladder filled with stones is wall echo shadow sign. Due to high echogenicity of anterior wall of gallbladder, superficial stones are visible while deeper stones and posterior gallbladder wall are not visible [9].

Among other techniques for the assessment of biliary injuries, biochemical testing of liver enzymes is a common clinical practice with a sensitivity of 90%. Any alteration in their value is a matter of concern for clinicians and builds an indication for investigation of underlying pathology. AST and ALT are generally considered as measures of hepatocellular damage. ALP levels are concerned with injury of biliary tract, while bilirubin level can be increased due to hemolysis or obstruction to flow of bile [10,11].

Another valuable parameter for the proper diagnosis and prognosis of gallstones is Complete Blood Picture [12,13]. It looks at the level of different types of blood cells, such as WBC, which might indicate infection [12].

In order to establish effective diagnostic clinical strategies for the early detection and prognosis of gallstones in Pakistan, it was important to understand the relationship among different laboratory parameters. It was the first research of its kind and no prior data are available on this subject. Our goal of study was to evaluate the relationship of ultrasonographic findings, hemolytic indices and liver function tests with gallstones.

## Methodology

It was a prospective study carried out in Civil Hospital Karachi (DUHS) and Liaquat National Hospital, two largest tertiary care hospitals of Karachi, Pakistan. Duration of study was from July 2011 to October 2012. The study was carried out on diagnosed, pre-operative and symptomatic patients of cholelithiasis. Diagnosis was based upon history, physical examination and ultrasound examination. Abdominal ultrasound was performed by expert sonologists who had experience of more than 5 years. All Ultrasounds were performed by two sonologists and all diagnoses were made by consensus. Informed written consent was taken from every patient or attendant of patient after full explanation of procedure regarding the study. Exclusion criteria were patients of gallbladder and pancreatic carcinoma, emergency operations, patients having age <12 years and non-cooperative patients, who refused to give written consent for participation in the study. Individuals who had stones in their gallbladder were selected randomly, without regard to age or gender. Total two tests were performed on each patient after diagnosis by ultrasonography. These were complete blood count and liver function tests. Complete blood picture include WBC count, RBC count, RBC morphology, Hematocrit, Neutrophil count, lymphocyte count, basophil count, blood urea nitrogen and serum creatinine. Liver function tests include total bilirubin, SGPT and alkaline phosphatase. Normal values for total leukocyte count are 5,000 to 10,000 WBC per cubic millimeter (mm3). Normal differential leukocyte counts are, Neutrophils 50%-75%, lymphocytes 25%-40%, basophils 0%-1%. Normal hematocrit values are 45% to 52% for men and 37% to 48% for women, hemoglobin is 11.5-16 millimoles/liter for men, 13–18 millimoles/litre for women, RBC count is 4.2 to 5.9 million cells/cmm. Noraml serum creatinine is 0.5-1.5 mg/dL for men and 0.6-1.2 mg/dL. Normal range for blood urea nitrogen is 7–20 mg/dL. Normal range for alkaline phosphatase is 30 to 136 IU/L, for total bilirubin is 0.1–1.0 mg/dL and for SGPT is 7 to 56 IU/L. Any value above or below normal was considered as abnormal finding. All the demographic data, laboratory findings and ultrasonographic features were noted in a structured Performa. For the proper evaluation of socioeconomic status, criteria were made according to guide lines of World Bank [14,15]. To make it simple the U.S Dollar was converted into Pakistani rupees and at that time 1 U.S dollar was equivalent to 93.4 Pakistani rupees and the result was rounded to nearest hundred.

Sample size was calculated by using Open-epi sample size calculator, prevalence (p) = 35% d = 5% and confidence interval (C.I) 95% = 350. Study was approved by Ethical Review committee of Civil Hospital Karachi, Dow University of Health Sciences.

All the data was entered and analyzed through SPSS 19. Mean and standard deviation were used for continuous data and percentage and frequency were calculated for categorical data. All the percentages and frequencies were calculated by considering n = 454

## Result

There were 454 diagnosed and pre-operative patients of gallstones presents in the study. There were 120(26.4%) males and 334(73.6%) females. Patients were between ages of 19 and 74 years and most of them were in their fourth decade of life with a mean age of 42.80 ± 12.26 years. Most of the patients belonged to low middle class 208(45.8%) followed by lower class 188(41.4%), higher middle class 46(10.1%) and higher class 12(2.6%) (Table 1).

**Table 1:** Table representing demographic, ultra sonographic and LFT variables

**Serial no**	**Variables**		**Frequency (n = 454)**	**Percentages (%)**
1	**GENDER:**			
		a) Male	120	26.4
		b) Female	334	73.6
2	**Socioeconomic classes**			
		a) High class	12	2.6
		b) High middle class	46	10.1
		c) Low middle class	208	45.8
		d) Low class	188	41.4
3	**Ultrasonographic changes of liver**			
		a) Normal parenchyma	362	79.7
		b) Hepatomegaly	92	20.2
4	**Fatty liver**			
		a) Yes	144	31.7
		b) no	310	68.3
5	**Number of gallstone**			
		a) Single	70	15.4
		b) Multiple	384	84.5
6	**Spleenomegaly**			
		a) Yes	16	3.5
		b) No	438	96.4
	**LIVER FUNCTION TEST**			
7	**Alkaline phosphatase**			
		a) Elevated	186	41.0
		b) Normal	268	58.9
8	**SGPT**			
		a) Elevated	160	35.2
		b) Normal	294	64.2
9	**Total bilirubin**			
		a) Elevated	62	13.6
		b) Normal	392	86.3

In ultrasonography all patients had a finding of Echogenic mass with shadowing, meaning that stones were present in 454(100%) patients. Most of the suspects had multiple stones 384(84.5%) while few had single stones 70(15.4%). Fatty liver was found to be present in 144(31.7%) patients and 92(20.2%) had hepatomegaly. Splenomegaly was present in 16(3.5%) patients (Table 1).

In liver function tests, total bilirubin was elevated in 62(13.6%) patients while 392 (86.3%) had normal value. Alkaline phosphatase was elevated in 186(41.0%) patients while SGPT was found to be raised in 160(35.2%) patients (Table 1).

In complete blood picture test, leukocytosis was found to be present in 114(25.1%). Neutrophil count was raised in 72(15.9%) followed by lymphocytes in 28(6.2%) and basophils in 32(7%). RBC count was increased in 28(6.2%) while hemoglobin was fall in 214(47.1%) patients (Table 2).

**Table 2:** Table represent variables regarding complete blood picture test

**Serial no**	**Variables**		**Frequency (n = 454)**	**Percentages (%)**
	**Complete blood count**			
1	**WBC count**			
		a) Normal	340	74.9
		b) Elevated	114	25.1
2	**Neutrophil count**			
		a) Elevated	72	15.9
		b) Normal	382	84.1
3	**Basophil count**			
		a) Elevated	32	7.0
		b) Normal	422	92.9
4	**Lymphocyte count**			
		a) Elevated	28	6.2
		b) Normal	380	83.7
		c) Below	46	10.1
5	**RBC count**			
		a) Elevated	28	6.2
		b) Normal	298	65.6
		c) Below	128	28.2
6	**RBC morphology**			
		a) Normochromic anisocytosis	66	14.5
		b) Normochromic normocytic	310	68.2
		c) Hypochromic anicocytosis	78	20.7
7	He**moglobin**			
		a) Elevated	4	0.9
		b) Normal	236	51.9
		c) Below	214	47.1
8	**Hematocrit**			
		a) Elevated	6	1.3
		b) Normal	226	49.8
		c) Below	220	48.5
9	**Blood urea Nitrogen**			
		a) Elevated	186	41.0
		b) Normal	256	56.4
		c) Below	12	2.6
10	**Serum creatinine**			
		a) Normal	378	83.4
		b) Elevated	46	10.1
		c) Below	8	1.8

Hematocrit level was low in 220(48.5%) people and the most common type of RBC morphology was found to be normochromic and normocytic in 310(68.2%) (Table 2).

Interestingly blood urea nitrogen was found to be raised in 186(41%) patients and serum creatinine was elevated in 46(10.1%) patients (Table 2).

Rest of the comparison of frequencies and percentages are given in tables.

## Discussion

Gallstones and cardiovascular diseases are common diseases worldwide and have considerable economical impact. Among gastroenterological diseases, GD is one of the world’s most expensive medical conditions. In the United States, there are more than 500,000 cholecystectomies, the total cost of which exceeds 5 billion dollars. Gall stones are considered as an avoidable cause of death [4,14]. It’s a fact that more than 95% disorders of biliary tract are due to cholelithiases [16]. Gallstones are seen in all age groups but the incidence increases with every decade of life and they were found to be most prevalent in 4th and 5th decade of life [5,8]. Incidence of gallstones was found many folds higher in females as compared to males and this increase was more during child bearing age. Our findings were also consistent with past studies [4,17]. These findings draw attention to the fact that in reproductive aged women, risk is 2–3 times higher as compared to non-reproductive age women. Reason for this increment is well understood now and it is due to elevated estrogen levels, which increase cholesterol excretion in bile by causing its super saturation with cholesterol [4].

In the present study efforts have been made to determine most common variety of stones present in gall bladder either single or multiple. According to our findings multiple stones were mostly present in patients of gallstones, which were in accordance with studies in the past [4,18]. Gallstones are of three varieties; most commonly they are composed of cholesterol followed by pigment and mixed stones [4].

It was indicated clearly in ultrasonographic findings that non alcoholic fatty liver had a significant association with gallstones; our findings were consistent with findings of the past studies [19-21]. This confirms the fact that due to fatty liver there is accumulation of lipids, specifically triglycerides in the hepatocytes, which triggers inflammatory responses that prompt the leakage of liver enzymes into the blood stream. Due to the fatty liver, gall bladder doesn’t empty normally, thus causing bile accumulation which precipitate gallstones.

Interestingly, it was found in our study that 3.5% patients of gallstones were also associated with Splenomegaly which has not been previously reported.

Long list of investigations, makes the diagnostic pathway complex and expensive but good clinical history has been worked out as the best predictor of gallstones. Abnormal liver function tests were most common in patients with gallstones. Raised Alkaline phosphatase has emerged as the most reliable predictor of gallstones after ultrasonography which was consistent with the findings indicated in the past [22-25]. In our study, subjects were predominantly females and increase in the level of alkaline phosphatase might be due to increased bone turn over or simultaneous formation of osteoid in these females [26].

Bilirubin also represented one of the indicators of gallstones but not as reliable as Alkaline phosphatase. It seems to be raised in 13.6% patients which was consistent with past studies [23-25,27-30]. Actually serum bilirubin is important in predicting postoperative/preoperative procedural outcomes. The degree of hyperbilirubinemia reflects the degree of liver dysfunction affecting both nutrition and reticuloendothelial cells [31].

Occurrence of gallstones was positively correlated with rise in SGPT levels. Our findings correlate with findings of past studies but frequency was found to be much higher as compared to the past [30,32,33]. It may be due to the fact that the study was published in 1994 and this reflects the change in trends of biochemical parameters. Our findings highlight the fact that due to gallstone disease liver is inflamed and damaged which simultaneously causes rise in hepatic enzymes in blood.

Blood tests play an important role in biliary tract diseases. It was found in our study that there was leukocytosis present in 1/4th patients, findings were consistent with past studies [30]. This reflects the fact that there was also role of certain bacterial infections which triggered the formation of gallstones as highlighted by rise in WBC count [34].

Principal finding of our study was the massive increment in blood urea nitrogen and it was increased in approximately 42% patients which were very high as compared to a study in Korea [35]. Literature concerning the relationship between gallstones and blood urea nitrogen is scarce and it was the first research which indicates the positive and significant relationship. Further researches will be necessary to figure out a better understanding of this relationship.

Another principal and interesting finding of our study was fall in hemoglobin level in nearly half of the subjects. It may possibly be due to the fact that bilirubin is the main constituent in the formation of gallstones. It is mainly formed by the breakdown of hemoglobin, which leads to low levels of hemoglobin in patients of gallstones.

This was the first prospective study dedicated to this subject in Pakistan. Limitations of this study were lack of comparison between pre and post operative biochemical and ultrasonographic parameters. Second limitation was short sample size and it did not represent the whole population; it was based only in the patients of two tertiary care hospitals of Karachi, Pakistan.

In our study, it was found that there were abnormalities in liver function and complete blood count test in patients of gallstone. In the light of these findings, it was recommended that all patients should go through the process of ultrasound and all the biochemical parameters should be analyzed before surgery. Special attention should be given to the abnormal findings of hemoglobin, blood urea nitrogen and creatinine in order to evaluate and make management methods for underlying causes. Our study opens the forum of discussions and should be continued in more advanced and modified phases. Further studies will be highly recommended on the basis of our findings.

## Conclusion

In conclusion, achievement in study of pathogenesis and physiology of gallstone diseases has allowed expanding indication for therapeutic treatment of gall bladder diseases and reducing the number of patients who undergo surgical treatment. From a public health point of view, it is not only important to study the background of gallstone formation but also explore demographic and biochemical markers related to the development of gallstones. If we are able to predict the contributing factors, then we can also prevent it by controlling those factors.

## Abbreviations

ALP: Alkaline phosphatase; CBP: Complete blood picture; LFTs: Liver function tests.

## Competing interests

Authors declared that they had no competing interests.

## Authors’ contribution

HMA and SS: Topic selection, analyzing and drafting manuscript. MME, HAS, JDK, MH and MS: data collection and critically reviewing manuscript. All authors read and approved the final manuscript.

## Authors’ information

HAFIZ MUHAMMAD ASLAM = final year student of Dow medical college, Dow university of health sciences

coolaslam8@hotmail.com

SHAFAQ SALEEM = final year student of Dow medical college, Dow university of health sciences. malaika_leo@hotmail.com

MUZZAMMIL EDHI = final year student of Liaquat national medical college, Karachi university.

muzzamil10@hotmail.com

HIBA ARSHAD SHAIKH = final year student of Dow medical college, Dow university of health sciences

hibashaikh_91@hotmail.com

JEHANZEB DANIAL KHAN = final year student of Liaquat national medical college, Karachi university

jdk_smackdown@yahoo.com

MEHAK HAFIZ = fourth year student of Liaquat national medical college, Karachi university creation.mg@gmail.com
